# Presynaptic DLG regulates synaptic function through the localization of voltage-activated Ca^2+^ Channels

**DOI:** 10.1038/srep32132

**Published:** 2016-08-30

**Authors:** César Astorga, Ramón A. Jorquera, Mauricio Ramírez, Andrés Kohler, Estefanía López, Ricardo Delgado, Alex Córdova, Patricio Olguín, Jimena Sierralta

**Affiliations:** 1 Biomedical Neuroscience Institute, Faculty of Medicine, Universidad de Chile, Santiago, Chile; 2 Program of Physiology and Biophysics, Institute of Biomedical Sciences, Faculty of Medicine, Universidad de Chile, Santiago, Chile; 3 Neuroscience Department, School of Medicine, Universidad Central del Caribe, Bayamon, 00956, PR, USA; 4 Department of Biology, Faculty of Science, Universidad de Chile, Santiago, Chile; 5 Program of Human Genetics, Institute of Biomedical Sciences, Faculty of Medicine, Universidad de Chile, Santiago, Chile.

## Abstract

The DLG-MAGUK subfamily of proteins plays a role on the recycling and clustering of glutamate receptors (GLUR) at the postsynaptic density. *discs-large1* (*dlg*) is the only DLG-MAGUK gene in *Drosophila* and originates two main products, DLGA and DLGS97 which differ by the presence of an L27 domain. Combining electrophysiology, immunostaining and genetic manipulation at the pre and postsynaptic compartments we study the DLG contribution to the basal synaptic-function at the *Drosophila* larval neuromuscular junction. Our results reveal a specific function of DLGS97 in the regulation of the size of GLUR fields and their subunit composition. Strikingly the absence of any of DLG proteins at the presynaptic terminal disrupts the clustering and localization of the calcium channel DmCa1A subunit (Cacophony), decreases the action potential-evoked release probability and alters short-term plasticity. Our results show for the first time a crucial role of DLG proteins in the presynaptic function *in vivo*.

Synaptic scaffolding proteins are essential for the formation, maintenance and remodeling of synapses by recruiting synaptic constituents like neurotransmitter receptors, adhesion proteins and ion channels^1^. The discs-large proteins (DLG) are a subfamily of the Membrane Associated Guanylate Kinase (MAGUK) widely expressed in the nervous system and conserved across the animal kingdom^2^. In vertebrates, there are four *dlg*-MAGUK genes; *dlg1* (*sap97/hdlg*), *dlg2* (*sap102*/*neuroendocrine discs large*), *dlg3* (*psd93*/ch*apsyn-11*0) and *dlg4* (*psd95*/*sap90*)^3^. Although all members of the DLG family are expressed in the post-synaptic density, their enrichment is variable. Notably, all of them are also expressed at different levels in the axon^4^, however, the *in vivo* role of DLG in presynaptic function has not been determined.

*dlg1* is the only gene of the DLG-MAGUK subfamily in *Drosophila*^5^. Similar to vertebrate genes, two forms of the gene are expressed as the result of two transcription start sites. DLGA (α form) and DLGS97 (β form) are distinguished by the inclusion of an L27 domain in beta forms located in the amino terminus of the protein^6^. In vertebrates DLG4/PSD95 is predominantly expressed as α form while DLG1/SAP97 is mainly expressed as β form. DLGA is expressed in epithelial tissues, somatic muscle and neurons; in turn, DLGS97 is not expressed in the epithelial tissue. In the larval neuromuscular junction (NMJ), a glutamatergic synapse, both *dlg* products are expressed pre and postsynaptically^7^. Hypomorphic *dlg* alleles display underdeveloped subsynaptic reticulum, bigger glutamate receptors fields and an increased size of synaptic boutons, active zones and vesicles^8^,^9^,^10^. Additionally to these morphological defects, altered synaptic responses such as increased excitatory junction currents (EJC)^9^ and increased amplitude of miniature excitatory junction potentials have been observed^7^. The strong morphological defects make difficult to distinguish developmental defects from the role of DLGs in the basal function of the mature synapse^9^,^11^,^12^. We have previously reported form-specific null mutant strains for DLGA (*dlgA*^*40.2*^) and DLGS97, (*dlgS97*^*5*^). These mutants do not show the gross morphological defects observed in hypomorphic mutants, although still show functional synaptic defects, supporting a role of DLG proteins in the mature synaptic function^7^.

Here, combining genetic, electrophysiology and immunostaining techniques we dissect the role of DLG proteins at the pre and postsynaptic compartments. Our results show the specific requirement of postsynaptic DLGS97 for normal glutamate receptor (GLUR) distribution. In turn, both DLG proteins increase the release probability by promoting voltage-dependent Ca^2+^ channel localization. Our results demonstrate for the first time a relevant role to DLG proteins in the presynaptic function contributing to Ca^2+^ mediated short-term plasticity and probability of release.

## Results and Discussion

### Isoform specific post-synaptic defects in *dlg* mutants

**Figure 1:**
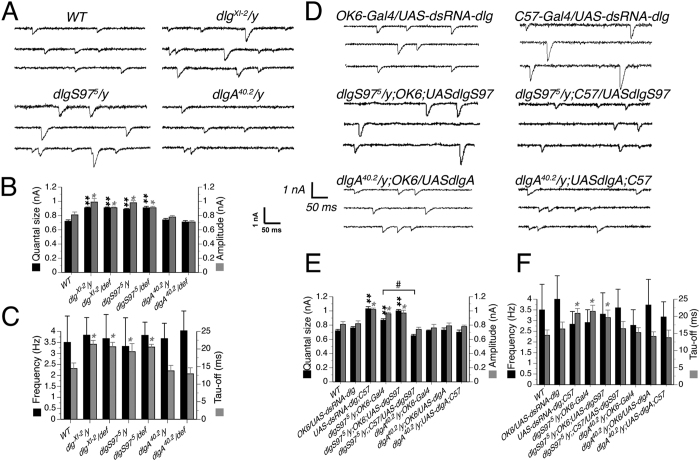
DLGS97 regulates quantal size and the time constant of the mEJC. (**A**) Representative traces of voltage clamp recordings of spontaneous synaptic currents observed in WT and *dlg* mutant larvae. (**B**) Quantification of the average (n = 6) quantal size and the average amplitude in WT, *dlg* mutants and *dlg* mutants over a deficiency covering the *dlg* locus (n = 6). The quantal size was obtained by plotting frequency histograms of the synaptic events recorded during 2 minutes for each genotype and fitted by a Log-Normal function, the quantal size was obtained from the pick of the fitted curve. Both the quantal size and the amplitude are significantly larger than WT only in *dlg* and *dlgS97* mutants. (**C**) Quantification of the average frequency and the average time constant of the decay (tau-off) of the spontaneous currents in *dlg* mutants and *dlg* mutants over a deficiency covering the *dlg* locus. The frequencies in WT and mutants are similar while, only in *dlg* and *dlgS97* mutants the time constants show difference with WT. (**D**) Representative traces of voltage clamp recordings of spontaneous synaptic currents observed in the genotypes described. (**E**) Quantification of the average quantal size and the average amplitude in WT and different genotypes. (**F**) Quantification of the frequency and average time constant of spontaneous currents recorded for the different genotypes (n = 6). *p < 0.05; **p < 0.01 respect to the WT. ^#^p < 0.01 significance respect to the mutant. Columns represent the average ± SEM.

We have previously shown that flies carrying the severe hypomorph *dlg1* mutant allele, *dlg*^*XI-2*^ and the isoform specific *dlgS97* null mutant displayed increased amplitude of the spontaneous excitatory postsynaptic (junctional) potential (mEJP) without changes in frequency^7^. In addition all mutants displayed a decreased quantal content as measured by evoked post-synaptic potentials. Here we explored the specific defects behind these results. To characterize the synaptic transmission in WT and *dlg* mutants, we recorded post synaptic currents in HL3.1 solution^13^ on muscles 6 or 7 of third instar male larvae of the various genotypes. Recordings of spontaneous excitatory postsynaptic currents (mEJC) were obtained after blocking the voltage activated sodium channels (Fig. 1). Thereafter, histogram distributions were constructed with the amplitudes of the miniature events and the quantal size was estimated by the peak value obtained adjusting a Log-Normal distribution in each genotype. It is worth to emphasize that finding a phenotype on *dlgA* or *dlgS97* mutants means that DLGA or DLGS97 proteins by themselves cannot replace DLG function.

We compared the average amplitude of spontaneous postsynaptic potentials and, supporting our previous results^7^, we found that the average amplitude of the mEJC of the mutants *dlg*^*XI-2*^ (0.99 ± 0.05 nA) and *dlgS97* (0.98 ± 0.03 nA) were significantly larger compared to the average amplitudes of the mEJC of Canton-S strain used as WT control (0.81 ± 0.04 nA) and of *dlgA* (0.78 ± 0.02 nA) specific mutant (Fig. 1A,B). The same result was obtained comparing the quantal size (Fig. 1B; WT: 0.72 ± 0.02; *dlg*^*XI-2*^: 0.91 ± 0.01; *dlgS97*: 0.89 ± 0.01*; dlgA:* 0.74 ± 0.02). None of the mutants showed a significant change compared to the WT in the frequency of the mEJC (varying between 2 to 4 Hz, Fig. 1C^7^). As an additional control, we recorded all mutants over a deficiency covering the *dlg* gene, finding similar results (Fig. 1B). These findings are in accordance with the idea that DLGS97 protein and not DLGA is necessary for the quantal size determination.

Bigger quantal size could be of pre or postsynaptic origin as the result of increased neurotransmitter (NT) content in vesicles or increased glutamate receptor field’s size respectively. First, to determine the pre or post-synaptic origin of this phenotype, we expressed a *UAS-dsRNA* construct that targets all *dlg* products, under the control of the motoneuron promoter OK6-GAL4 or the muscle promoter C57-GAL4. As expected for a post-synaptic defect, the increased quantal size observed in *dlgS97* mutants was mimicked only by the decrease of DLG in the muscle (Fig. 1D,E; presynaptic dsRNA: 0.76 ± 0.02 quanta; postsynaptic dsRNA: 1.03 ± 0.03 quanta). The specific role of DLGS97 in the muscle is supported by the rescue of the *dlgS97* mutant phenotype only by the expression of DLGS97 in the muscle and not in the motor neuron (Fig. 1D,E). We looked at the effect of GAL4 expression in the mutant background in all experiments; neither of the GAL4 lines without the specific UAS constructs changed the phenotype of the mutants (Fig. 1E and data not shown). Again, none of the genotypes studied displayed differences with the WT in the frequency of the minis.

**Figure 2:**
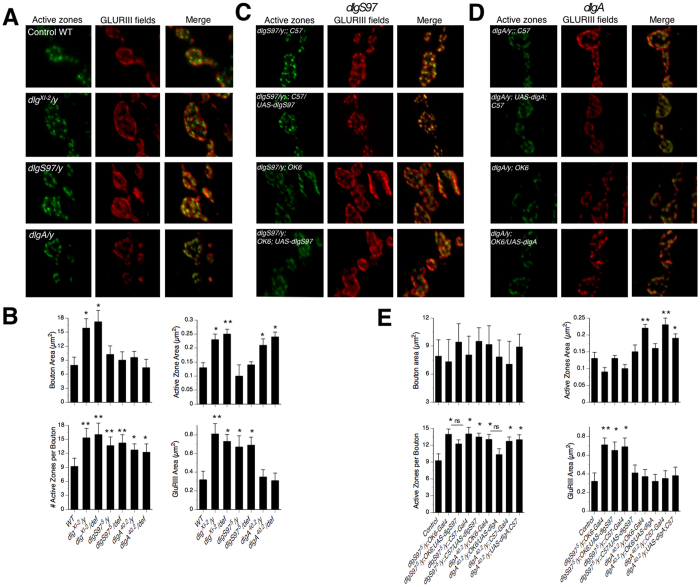
DLG proteins regulate the number of active zones per bouton but only DLGS97 regulates the size of the glutamate receptors fields. Confocal images of synaptic boutons labeled with antibodies against the active zone protein Bruchpilot (green) and antibodies against the constitutive subunit of the Glutamate receptor GLURIII (red). (**A**) WT and *dlg* mutants representative images. *dlg*^*XI*-2^ and *dlgS97* mutants show increased size of the GLUR-fields. (**B**) Quantification of the average size of the boutons (top-left), the average size of the active zones (top-right), the average number of active zones per bouton (bottom-left) and the average size of the GLUR-fields (bottom-right) in *dlg* mutants and *dlg* mutants over a deficiency covering the *dlg* region in the genome. (**C**) Representative images of motoneuron driver (GAL4-OK6) or the muscle driver (GAL4-C57) in the *dlgS97*^*5*^ mutant background with or without (genetic mutant control) expression of UAS-DLGS97. The size of the GLUR fields is rescued in *dlgS97* mutant by the expression of DLGS97 in the muscle but not in the neuron. (**D**) Representative images of motoneuron driver (GAL4-OK6) or the muscle driver (GAL4-C57) in the *dlgA*^*40.2*^ mutant background with or without (genetic mutant control) expression of UA-DLGA. (**E**) Quantification of the average size of the boutons (top-left), the average size of the active zones (top-right), the average number of active zones per bouton (bottom-left) and the average size of the GLUR-fields (bottom-right) in *dlgS97*^*5*^*and dlgA*^*40.2*^mutants with or without the expression of DLGS97 or DLGA in the neuron and in the muscle. The size of the GLUR fields is only restored to a WT size in *dlgS97* mutant by the expression of DLGS97 in the muscle but not in the neuron. The presynaptic expression of DLG is able to rescue the number of active zones per synaptic bouton, however only the expression of DLGA in the neuron is able to rescue the size of the active zones. Columns represent the average ± SEM n = 6. *p < 0.05, **p < 0.01 respect to the WT.

Changes in quantal size of postsynaptic origin could be due to higher number of post-synaptic receptors and/or a different composition of the postsynaptic receptors. An increase in the size of glutamate receptors fields has been described in *dlg* hypomorphic alleles including *dlg*^*XI-2*^ mutants^14^,^15^,^16^, in addition we previously showed that *dlgS97* but not *dlgA* mutants display bigger receptor fields^7^. Therefore, we compared the size of the glutamate receptor fields among the mutants and with WT and also measured the active zones using antibodies for the active zone protein Bruchpilot. Consistently with our previous results we found bigger glutamate receptors fields compared to WT only in *dlg*^*XI-2*^ and *dlgS97* mutants but not in *dlgA* mutants (Fig. 2A,B second row). Surprisingly, we also found an increased number of active zones per bouton in all mutants, a phenotype usually associated with an increase in the frequency of minis that we did not observe (Fig. 2B second row). In addition, we found an increased active zone area in *dlgA* and *dlg*^*XI-2*^ mutants (Fig. 2B first row).

As expected for a postsynaptic defect, the bigger size of the glutamate fields in *dlgS97* mutants was rescued by the expression of DLGS97 in the muscle but not by its expression in the motor neuron (Fig. 2C,E second row). These results confirm that DLGS97, but not DLGA is responsible for the regulation of the size of the receptors fields in the muscle.

The strict requirement of DLGS97 in the regulation of the size of GLUR fields supports results that have involved other DLGS97 interacting proteins in the regulation of the size of the glutamate receptors fields. METRO, an MPP-like MAGUK protein, has been shown to form a complex with DLGS97 and LIN-7 through the L27 domains present in each of the three proteins. *metro* mutants display decreased DLGS97 at the synapse and larger GLUR receptors fields than WT, even bigger than *dlgS97* mutants^16^. METRO and DLGS97 depend on each other for their stability on the synapse, thus, in *dlgS97* mutants, METRO and dLIN-7 are highly reduced at the synapse^16^. The similar post-synaptic phenotype of *metro* and *dlgS97* and the reported interaction between these two proteins allow us to propose that the increase size of GLUR fields is consequence of the loss of METRO due to the loss of DLGS97 protein^16^.

**Figure 3:**
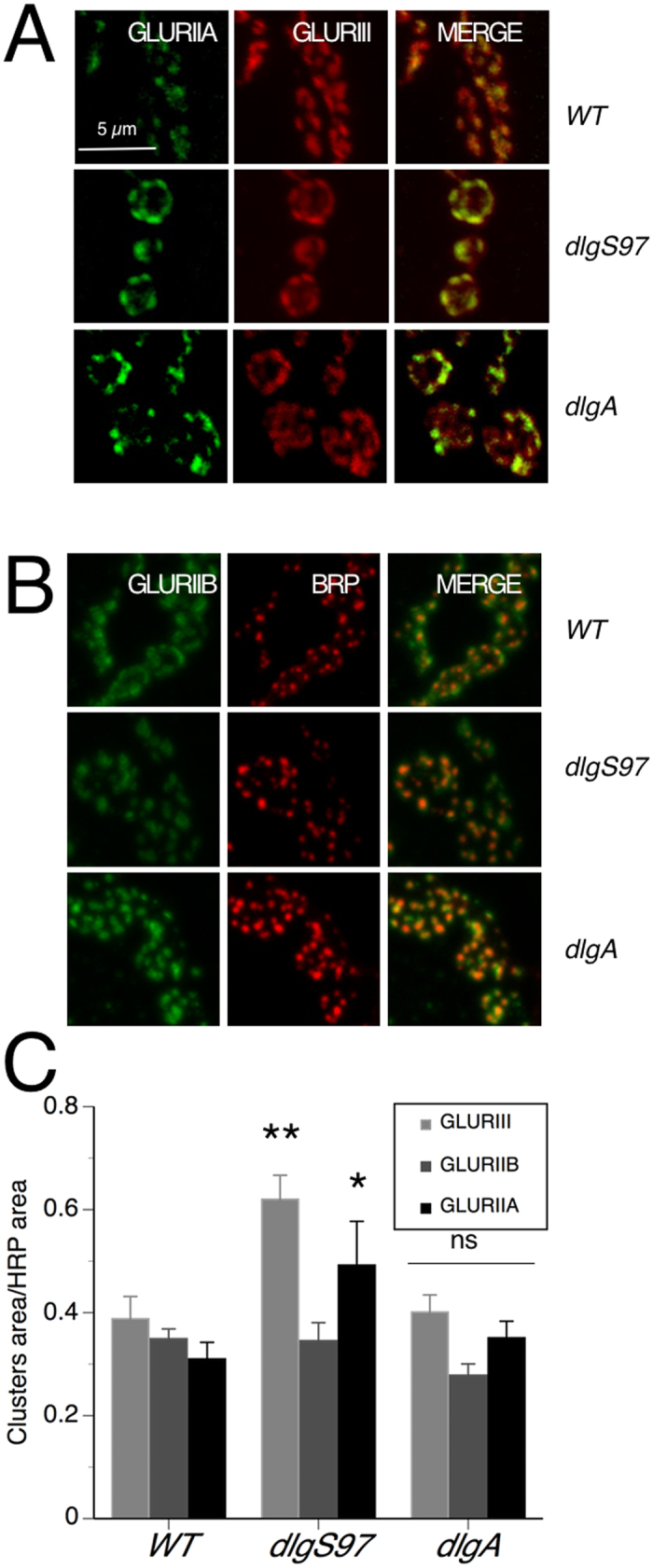
DLGS97 does not change the size of the GLURIIB receptor fields but increases the size of GLURIIA receptor fields. Confocal images of synaptic boutons of WT and *dlg* mutants labeled with antibodies against GLURIIA (**A**) or GLURIIB (**B**) in green and GLURIII (**A**) or the active zone protein Bruchpilot (**B**) in red. (**C**) Quantification of the size of the clusters for the three GLURs. *dlgS97* mutants display increased size of GLURIII and GLURIIA receptor fields. None of the mutants display changes in the size of GLURIIB-fields. Columns represent the average of 5 or 6 different larvae. *p < 0.05, **p < 0.01 respect to the WT.

As stated before, changes in quantal size of postsynaptic origin can also reflect a different composition of the receptors. *Drosophila* NMJ GLUR receptors are tetramers composed by obligatory subunits and two alternative subunits, GLURIIA and GLURIIB. Receptors composed by one of these two subunits differ in their kinetics; GLURIIB receptors desensitizes faster than GLURIIA receptors^17^,^18^,^19^. Thus, the kinetic of the spontaneous currents (mEJCs), is associated to the relative abundance of these two types of receptors in the GLUR fields. It has been shown that the abundance of GLURIIB but not of GLURIIA in the synapse is associated with the expression of *dlg*^20^. To analyze if *dlg* mutants display a change in the composition of the subunits abundance relative to the control we studied the kinetics of the mEJCs. Kinetics analyses of the mEJCs revealed that only *dlgS97* and the double mutant display a slower kinetic in the off response (see tau values in Fig. 1C,F), which is compatible with a different composition of the glutamate receptors fields regarding the proportion between GLURIIA and B receptors. The value of tau also increased in larvae expressing *dsRNA*-*dlg* in the muscle, but not by its expression in the motor neuron (Fig. 1F). Finally tau-off values recovered the WT value only with the expression of DLGS97 in the muscle (Fig. 1F). As we observed slower mEJCs, our results suggest an increase in the ratio of GLURIIA/GLURIIB. It is known that receptors containing the GLURIIA subunit display bigger conductance and slower inactivation kinetics than receptors containing the GLURIIB subunit. Thus, synapses with post-synaptic receptors fields containing proportionally less GLURIIB subunits would display bigger and slower mEJCs^17^ similar to the phenotype observed in *dlgS97* mutants. To confirm this hypothesis, we evaluated the abundance of GLURIIA and GLURIIB receptors by immunofluorescence in the NMJ of WT and *dlg* mutant larvae. The immunofluorescence that allowed the detection and quantification of GLURIII and GLURIIB fields was performed with paraformaldehyde (PFA) fixative. However, the immunofluorescence to detect GLURIIA receptors only works fixating the tissue with Bouin reagent. Thus, in order to be able to compare between these two fixations, we normalized the size of the GLUR fields by the HRP staining that labels the whole presynaptic bouton. First, as a control, we double stained GLURIIA and GLURIII in the same larvae. Our results show that, in agreement with the results in Fig. 2 using PFA fixative, GLURIII fields display bigger size only in *dlgS97* mutants and not in *dlgA* mutants (Fig. 3A,C). Even more, as predicted from the kinetic data, only *dlgS97* and not *dlgA* mutants display bigger GLURIIA fields while there are not difference in the size of GLURIIB fields between WT and the mutants (Fig. 3). Additionally our results show no difference in the number of GLURIIA or GLURIIB clusters between WT and *dlg* mutants (data not shown). Our immunohistochemical results confirm the prediction from the electrophysiological data revealing that in *dlgS97* mutants, GLURIIA subunits are proportionally more abundant in GLUR fields than in control larvae. In conclusion, our results show that *dlgS97* mutants display larger quanta and mEJCs with slower kinetic establishing its participation in the regulation of the size of GLUR fields where the increased size is obtained mainly through the recruitment of receptors containing GLURIIA subunits. A similar result was obtained by Chen and Featherstone^20^ who observed that the loss of GLURIIB receptors in the NMJ of *dlg*^*XI-2*^ mutant embryos, these observation suggest that either of the two DLG proteins are necessary for the localization of GLURIIB in the synapse but only DLGS97 is actively limiting the size of the clusters by regulating the number of GLURIIA receptors.

Taking into account previous reports that show the regulation of the synaptic localization of DLG by CAMKII^21^, the regulation of the subunit composition by CAMKII^22^ and these results, we could propose a mechanism by which, after a strong activation of CAMKII, the phosphorylation of DLGS97 would detach it from the synapse allowing the increase of the size of the GLUR fields (mechanism proposed by Koh *et al*.^21^ for DLG) by the recruitment of GLURIIA over GLURIIB. These changes should increase the synaptic response by two different mechanisms.

### Evoked responses unveil presynaptic defects in *dlg* mutants

**Figure 4:**
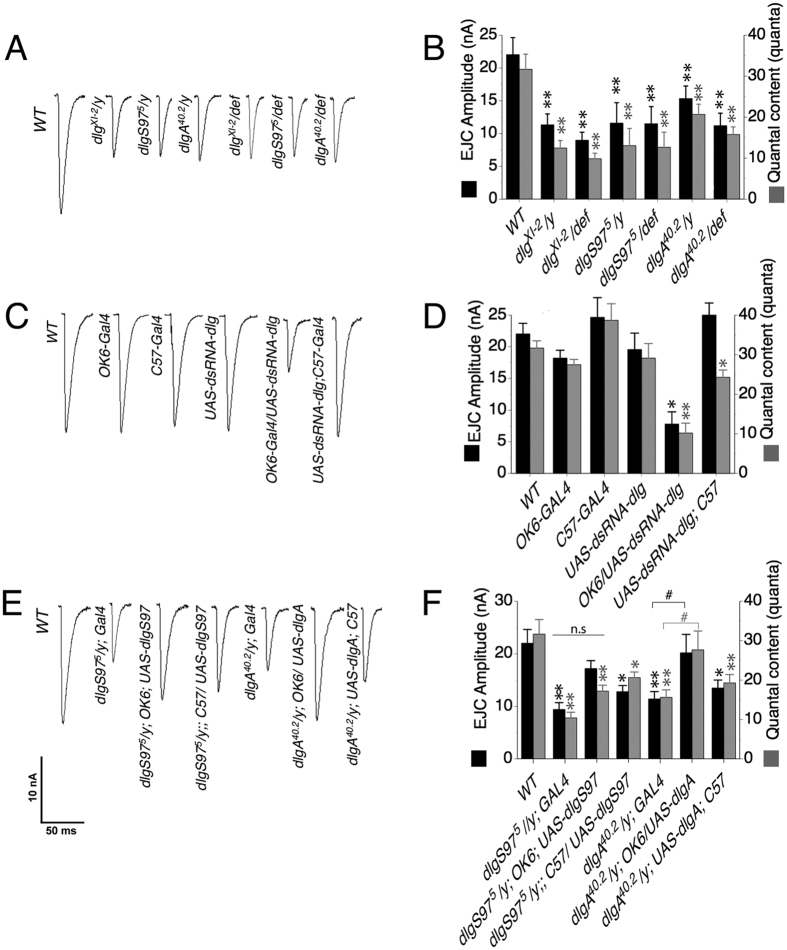
All *dlg* mutants display smaller evoked junction currents. (**A**) Representative traces of evoked synaptic current recordings of WT and *dlg* mutant larvae. Currents were recorded under voltage clamp with a holding voltage of −80 mV. (**B**) Quantification of the average current amplitude and the quantal content of WT, *dlg* mutants and *dlg* mutants over a deficiency that covers the *dlg* gene. All mutants show smaller currents and quantal content. (**C**) Representative traces of evoked synaptic current recordings of WT, GAL4-expressing larvae and larvae carrying *UAS-dsRNA-dlg* used as control as well as larva expressing *dsRNA-dlg* pre (OK6) or postsynaptically (C57), notice that only the expression of *dsRNA-dlg* presynaptically mimics the *dlg* phenotype. (**D**) Quantification of the average synaptic current amplitude and the average quantal content of the different genotypes in (**C**). (**E**) Representative traces of evoked synaptic current recordings of WT, GAL4 drivers in *dlg* mutant background alone or with UAS-DLGA or UAS-DLGS97 expressed pre or postsynaptically. (**F**) Quantification of the average current amplitude and the average quantal content of the genotypes shown in (**E**) Notice that only DLGA expressed presynaptically is able to completely rescue the *dlgA* mutant phenotype, while DLGS97 expressed pre and postsynaptically is able to rescue partially *dlgS97* phenotype. Columns represent the average ± SEM n = 6 larvae in average 20 responses per larvae. *p < 0.05, **p < 0.01 respect to the WT. Significant respect to the mutant control.

To determine if DLG proteins modulate the presynaptic release probability, we recorded excitatory junction currents (EJC) in the muscle by stimulating the nerve at 0.5 Hz in low extracellular Ca^2+^ (0.2 mM), both conditions to avoid synaptic depression. For all mutant genotypes the average peak amplitude and quantal content (EJC amplitude/quantal size) of the evoked responses were significantly smaller than WT (Fig. 4A,B). In congruence with our previous results^7^, the lower amplitude of the current response is accompanied by a decrease in the quantal content (Fig. 4B). Taking into account our results on the size of the GLUR fields in the mutant’s muscles, these results are compatible with a reduction of the neurotransmitter release in *dlg* mutants. A decreased neurotransmitter release could be associated with a decreased number of release sites in the boutons. However, as shown in Fig. 2A,B (second row) and in previous reports^9^, the number of active zones per bouton is increased in all *dlg* mutants with bigger active zones in *dlg*^*XI-2*^ and *dlgA* mutants (Fig. 2B first row).

The decrease in the evoked response could be a consequence of the absence of the specific form of DLG in the postsynaptic side, transmitted by unknown mechanisms or, alternatively, it could be the result of an effect of DLG on the probability of release. In order to explore where this phenotype originates (pre or post-synaptically) we downregulated DLG levels by expressing *dsRNA* against all forms of *dlg*. Compatible with a presynaptic defect, the expression of *UAS-dsRNA-dlg* presynaptically decreases the amplitude of the evoked response (Fig. 4C,D) while the same construct expressed postsynaptically using C57 promoter did not changed the amplitude of the EJCs (7.8 ± 1.92 nA vs. 25 ± 1.9 nA; presynaptic vs. postsynaptic expression, Fig. 4C,D). The presynaptic expression of the *dsRNA-dlg* also mimics the reduction in quantal content of the mutants, displaying a severe reduction in this parameter (Fig. 4C,D; WT: 31.67 ± 2.7 quanta; *OK6/UAS-dsRNA*: 10.2 ± 2.5 quanta, p = 0.0007). On the other hand, the postsynaptic expression of the dsRNA-dlg associates to a moderate but significant decrease on the quantal content, as expected from the effect already reported of the postsynaptic *dsRNA-dlg* on the quantal size and the lack of effect on the amplitude of the EJCs (*UAS-dsRNA; C57*: 24.27 ± 1.84 quanta, p = 0.047). The presynaptic effect of DLG is supported further by the rescue experiments. Thus, the amplitude of the evoked response and the quantal content in *dlgA*^*40.2*^ mutant is completely rescued by the selective expression of DLGA in the presynaptic compartment but not by its expression in the postsynaptic compartment (22.2 ± 2.5 nA and 13.5 ± 1.5 nA respectively, Fig. 4E,F). The pre-synaptic expression of DLGS97 improves the synaptic function increasing the average size of the EJCs such that the difference between the WT and the presynaptic-rescue is not significant (p = 0.49), suggesting a complete rescue. However, the average EJC in the presynaptic rescue is not different either from the control mutant animal (p = 0.056), which we interpret as the rescue not being complete and thus we use the term partial rescue. *DLGS97* does not, however rescued at al the quantal content. This is explained because although the amplitude of the current increased, the quantal size remains unchanged by the presynaptic expression of DLGS97. In consequence the quantal content does not increase as much as the current. On the other hand, the postsynaptic expression did not increase the amplitude of the evoked current. However, since it does rescue the quantal size (Fig. 1) the quantal content augmented enough to be different from the mutant control (p = 0.042; Fig. 4E,F). Notably, DLGA expressed presynaptically in *dlgA*^*40.2*^mutants not only rescued the EJC amplitude but also the number of active zones per bouton and the size of the active zones (Fig. 2D,E). On the other hand DLGS97 expression only partially rescued the increased number of active zones in *dlgS97*^*5*^ mutants (Fig. 2D,E). These results support a role of DLG proteins in the presynaptic function where DLGA seems to regulate more aspects than DLGS97. Despite the fact that both forms of DLG share most of their protein domains, neither of the two-forms is able to fully rescue the absence of the other, suggesting that both of them participate in a complex. The binding between the SH3 and GUK domains of MAGUK proteins has been described; this interaction (at least *in vitro*) is able to form intra or intermolecular associations and offers a mechanism by which DLGA and DLGS97 proteins could be associated to recruit proteins to a complex^23^,^24^.

Changes in the overall quantal content at these synapses may reveal presynaptic defects. However, genetic background and other independent modification could alter apparent release. To independently scrutinize alteration in the presynaptic release probability we look at two presynaptic properties, the short-term plasticity and the calcium dependency of quantal release.

### Short-term plasticity protocols reveal calcium entrance defects in *dlg* mutants

**Figure 5:**
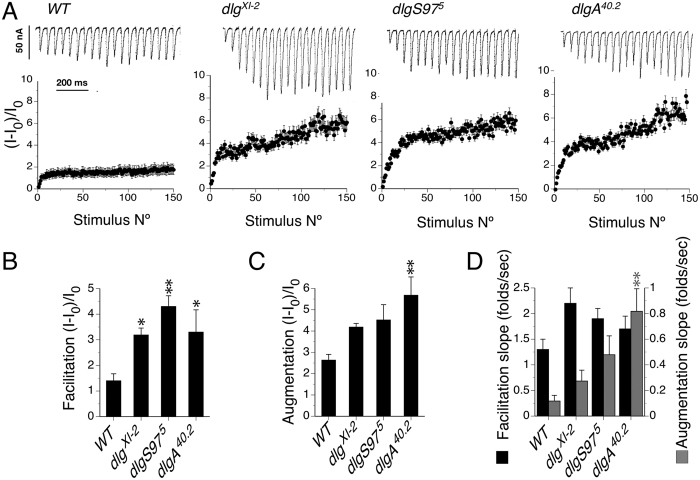
Short-term plasticity is altered in all *dlg* mutants. (**A**) Top, extract of representative traces of evoked synaptic currents showing the beginning of the responses to 150 stimuli at 20 Hz; bottom, average of the responses of WT and *dlg* mutants normalized to the size of the average responses at low frequency stimulation. Note the increased response of all mutants during the high frequency stimulation. (**B**) Quantification of the tetanic facilitation^43^. All mutants display an increased facilitation. (**C**) Quantification of the tetanic augmentation (the intersection of the line fitted for the second slope) for all genotypes. Only *dlgA* mutant show a significant difference in the values of the augmentation. (**D**) Quantification of the facilitation and augmentation slopes for all genotypes. Again only *dlgA* mutants display significant difference. The average response pre-tetanus is calculated with 5 stimuli at 0.5 Hz. Columns represent the average ± SEM n = 6 larvae. *p < 0.05, **p < 0.01 respect to the WT.

To explore the EJC phenotype observed in *dlg* mutants we carried out stimulation paradigms that allow us to characterize aspects of the short term plasticity that are known to depend on presynaptic functionality and give us clues about the mechanisms involved in the observed defects. First, we studied the response of the mutants to high frequency stimulation, 150 stimuli at 20 Hz. WT responses at high frequency stimulation show a fast increase in the amplitude of the response that then slows down (Fig. 5A). The fast initial increase is called facilitation and the second phase with smaller slope is called augmentation^25^. The time constant of the facilitation is believed to reflex the calcium dynamics in the terminal and its slope to be the product of the accumulation of calcium and the consequent calcium dependent increase in the probability of release^26^. The fractional increment in the mutants’ responses showed an increased facilitation in all mutants (Fig. 5B), while an increased augmentation was only significant in *dlgA* mutants compared to WT (Fig. 5C). Additionally all mutants showed a trend toward steeper slopes than WT, but only the augmentation slope in *dlgA* mutants reached statistical significance (Fig. 5D). These results support that the mutants display a lower probability of release than WT, which could reflect defects in the calcium dynamics or in the response to calcium.

Previous work in *dlg* mutants did not report defects in short-term plasticity^9^. These works differ from this one in methodological aspects, mainly that they were carried out in a media with high concentration of magnesium (20 mM) and calcium (1.5 mM). Our work was carried out in a media containing low magnesium (4 mM) and calcium (0.2 mM) concentration. It is known that magnesium reduces neurotransmitter release, probably due to partial blockade of VGCC^27^,^28^. Additionally, magnesium permeates more than sodium and potassium through GLURs^27^.

**Figure 6:**
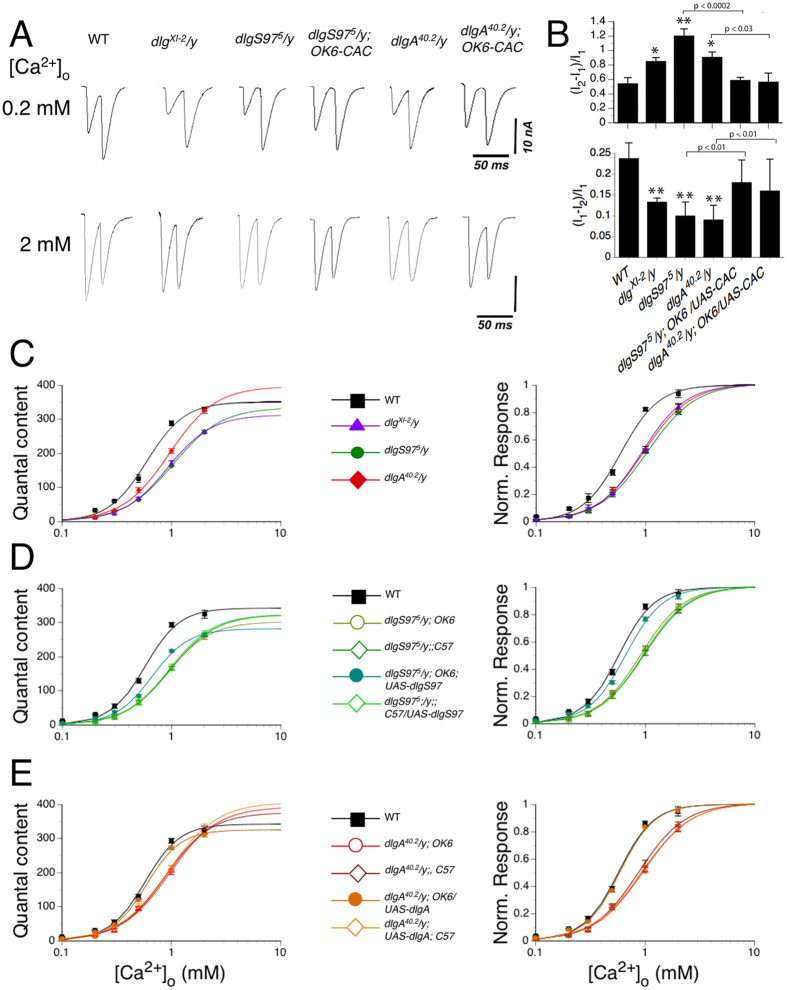
d*lg* mutants display defects in pair pulse facilitation and in the calcium dependency. (**A**) Representative traces of synaptic current recordings at 0.2 mM or 2 mM extracellular calcium concentration for WT, *dlg* mutants and CAC1 expressing *dlg* mutants in response to two stimulus separated by 20 ms. (**B**) Top: quantification of the pair pulse facilitation. The responses are normalized to the first pulse^43^. All mutants display increased facilitation that is rescued by the presynaptic expression of Cacophony, Bottom: quantification of the pair pulse depression. The responses at this calcium concentration are normalized to the first pulse^44^. All mutants display decreased depression that is rescued by the presynaptic expression of Cacophony. Panels (C–E) left represent the average quantal content of the evoked synaptic current responses at different extracellular calcium concentrations for WT and the different *dlg* genotypes. The panels on the right present the data normalized to the maximal value for each genotype calculated by the curve fitting shown in the left. (**C**) Average quantal content of the evoked synaptic current responses at different extracellular calcium concentrations for WT and *dlg* mutants. (**D**) Average quantal content of the evoked synaptic current responses at different extracellular calcium concentrations for the motoneuron driver (GAL4-OK6) or the muscle driver (GAL4-C57) in the *dlgS97*^*5*^ mutant background with or without (genetic mutant control) expression of UAS DLGS97. (**E**) Average quantal content of the evoked synaptic current responses at different extracellular calcium concentrations for the motoneuron driver (GAL4-OK6) or the muscle driver (GAL4-C57) in the *dlgA*^*40.2*^ mutant background with or without (genetic mutant control) expression of UAS DLGA. Each data in (**B**–**D**) corresponds to the average ± SEM of six larvae with at least 20 synaptic responses recorded. *p < 0.05, **p < 0.01 respect to the WT.

To better evaluate the calcium dynamics in the terminal we carried out pair pulse (PP) experiments (Fig. 6A,B), a well-known paradigm to evaluate presynaptic calcium dynamics. In PP, a second depolarization shortly after the first one carried out in low extracellular calcium concentration elicits an increased release of neurotransmitter thought to reflect the increased calcium concentration in the terminal reached after the first stimulus. According to this, and posing as the working hypothesis that DLG affects presynaptic calcium dynamics, we should expect that a second pulse would increase the release in a bigger proportion, since the first stimulus did not release much of the ready releasable pool. Conversely, a second stimulus given at high calcium concentration produces a decrease in the release of neurotransmitter, which is considered to originate in the partial depletion of the ready releasable pool at the release sites^26^. Thus, a second pulse at high calcium concentration should elicit a smaller decrease of the release since an inferior entrance of calcium should produce less depletion of the ready releasable pool of vesicles.

Consistently with a decreased calcium entrance, all mutants displayed increased pair pulse facilitation at low calcium concentration and decreased pair pulse depression at high calcium concentration (Fig. 6A,B). These results support a defect in the calcium entrance to the terminal as the underlying defect in *dlg* mutants causing the evoked stimuli defects. To characterize the calcium dependency of the release in the mutants we measured the evoked responses at different calcium concentrations (Fig. 6C). It can be observed that for all the mutants and at most calcium concentrations, the quantal content of the evoked response is lower than the control (Fig. 6C left). The only exception is seen at 2 mM calcium where the quantal content of the *dlgA* mutants and the control are not different to each other (Fig. 6C left). However, even at this calcium concentration the quantal content of *dlgS97* and the double mutant *dlg*^*XI-2*^ are significantly lower than the control. To get insight about the release process we fit the responses to a Hill equation. This type of fitting better estimate the maximum responses and the EC50, which is masked in the overall release of different backgrounds. This is observed in the graph with the normalized responses by the maximal quantal predicted. The adjusted curves show that mutants reach the theoretical maximal quantal content at higher calcium concentration than the WT and that the EC50 for the mutants is diminished respect to the WT (Figs 6C and S2). To confirm the presynaptic origin of the defect in the calcium dependency, we carried out the calcium dependency in the mutant genotypes expressing DLGA or DLGS97 pre or postsynaptically (Fig. 6D,E). The quantal content analysis shows that only the presynaptic expression of DLGA in *dlgA* mutants completely rescued the calcium dependency, in line with our previous results that show the importance of DLGA in the presynaptic compartment (Figs 6E and S2). On the other hand the presynaptic expression of DLGS97, although it rescued the calcium dependency, failed to rescue the maximal quantal content (Figs 6D and S2). Observing the graph with the normalized responses, DLGA as well as DLGS97 both are able to restore the WT calcium dependency (Fig. 6D,E left panels). The inability to rescue the maximal quantal content could be explained by the existence of synaptic compensatory mechanisms that allow to counterweigh the bigger quantal size in *dlgS97* mutants, which we showed before that is not rescued by the presynaptic expression of DLGS97.

Facilitation is thought to depend on the resultant of the calcium entrance, calcium release from intracellular stores and the clearance of cytosolic calcium. So, the defects in facilitation observed in the mutants could be due to a decreased calcium entrance but also they could be due to a defect on the clearance of calcium. In a preliminary experiment we measured the relative changes of the total intracellular calcium concentration in the bouton using the genetically encoded calcium indicator GCamp6f. As shown in Fig. S1, GCamp6f expressed in control flies (*OK6-GAL4/UAS-GCamp6f*) respond with a fast and transitory change in the cytoplasmic calcium of the boutons when they are exposed to a local pulse of potassium. The same experimental approach in *dlgS97* mutant larvae (*dlgS97*^*5*^*/y; OK6-GAL4/UAS-Gcamp6f*) reveals that the rise of the calcium response is significantly slower than the control; additionally the recovery of the response is also significantly slower (Fig S1). These preliminary experiments suggest a defect in calcium entrance in the mutants but they also support a defect in the extrusion that hint to additional defects. Further experiments are needed to clarify the calcium kinetics involved since these experiments were measuring the bulk of calcium change and in doing this approximation, we are losing the nanodomain changes that are known to be the ones that regulate the neurotransmitter release^29^.

### *dlg* mutants show altered distribution of voltage gated calcium channels at the synapse

**Figure 7:**
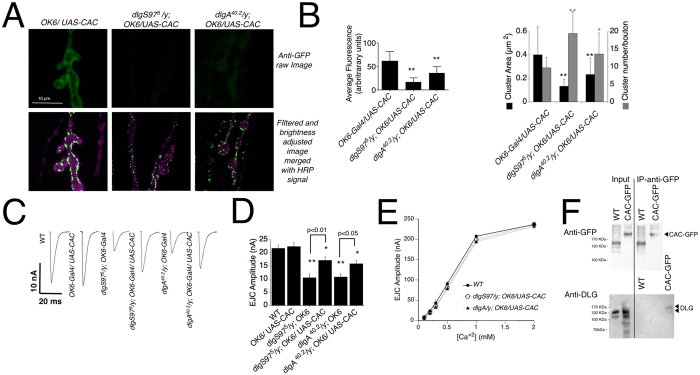
Levels and localization of Cacophony channel at the synapse depend on the presence of DLGA and DLGS97. (**A**) Representative confocal images of WT and *dlg* synaptic boutons expressing CAC1. The upper raw are images without any manipulation, note the very low signal observed in the mutants. The second row presents images that were filtered and adjusted in order to see the clusters, note that they are smaller and not always strictly associated to the periphery of the synaptic bouton. (**B**) Quantification of the average fluorescence signal, number of clusters per synaptic bouton and the average area of the clusters in WT and *dlg* mutants. (**C**) Synaptic currents recordings at 0.2 mM [Ca^2+^] in WT and WT and *dlg* mutants expressing CAC1. (**D**) Quantification of the synaptic current amplitudes of the genotypes showed in **(C**), (**E**) EJC average amplitudes in WT and *dlg* mutants expressing CAC1 at different extracellular calcium concentration from 0.2 to 2 mM. Compare with Fig. 6C. (**F**) Immunoprecipitation assay using anti-GFP antibody and detecting DLG with anti-DLG_PDZ_. Homogenized heads of WT or ELAV-GAL4/UAS-CAC1-GFP flies were incubated with anti-GFP antibody conjugated to magnetic beads. The proteins precipitated were run in an SDS page and transfer to nitrocellulose to be blotted with anti-DLG antibody. In parallel half of the sample was run in the same gel and blotted with anti-GFP antibody. The blot with anti DLG does not show more bands than the ones shown in the figure, however the blot with GFP antibodies shows several unspecific bands. Columns represent the average ± SEM, n = 6 larvae. *p < 0.05, **p < 0.01 respect to the control.

Since the results described above including the calcium dependency of the release as well as the parameters of the short-term plasticity suggest that the calcium entrance to the terminal is impaired, a view that is supported by the preliminary data measuring the cytosolic calcium, we focus the next experiments on the calcium entrance. The main calcium entry to the terminal is the voltage gated calcium channel (VGCC) encoded by the *Drosophila* gene *cacophony*^30^. We took advantage of a UAS-cacophony1-EGFP transgenic fly (CAC-GFP)^31^ to study the distribution of the channel in WT and mutant genotypes. CAC-GFP overexpressed in WT background localizes in the synapse in a strictly plasma membrane-associated manner in big clusters closely associated with release sites (Fig. 7A^31^). However, CAC-GFP overexpressed in *dlgS97* or *dlgA* mutant background displays a significant decrease in the expression accompanied by a more disperse localization with significantly smaller clusters, suggesting that the Cacophony protein might not be properly delivered or anchored to the plasma membrane in *dlg* mutants (Fig. 7A,B).

We reasoned that if *dlg* mutants had a defect on calcium entrance, the over expression of calcium channels should rescue at least partially the phenotype. We took advantage of the fact that CAC-GFP construct encodes a functional channel and recorded from control and *dlg* mutants overexpressing CAC. As expected and supporting a decreased calcium entrance in the mutants, *dlgS97* and *dlgA* mutants that overexpress CAC-GFP display significantly bigger evoked EJCs compared to *dlg* mutants (Fig. 7C,D), without a change of phenotype in the spontaneous currents (WT: 0.7 ± 0.016 nA, *dlgS97*^*5*^: 0.98 ± 0.12 nA, *dlgA*^*40.2*^: 0.73 ± 0.02 nA, compare with Fig. 1A,B). Additionally, the over expression of CAC-GFP partially rescued the pair pulse facilitation and the pair pulse depression (Fig. 6A,B) as well as the calcium curve (Fig. 7E).

The disrupted localization of CAC could result from the disturbance of a normal direct association to DLG or it could be affected indirectly. To test this we carried out an immunoprecipitation assay using flies that express CAC-EGFP in all neurons. As observed in Fig. 7F, antibodies against GFP were able to precipitate DLG together with Cacophony-GFP, supporting that Cacophony channel is part of the DLG complex in the boutons.

A possible interaction between DLG and VGCCs is the VGCC auxiliary subunits. The α_2_δ auxiliary subunit (Straightjacket in *Drosophila*) increases calcium channel activity and plasma membrane expression of CaV2 α_1_ subunits^32^ and Cacophony^33^. The β auxiliary subunit increases plasma membrane expression of several mammalian VGCC classes^34^. Intriguing β subunits are also MAGUK proteins and they are able to release the VGCC α subunit from the endoplasmic reticulum retention. It may be speculated that DLG through their SH3-GUK domain might be playing the role of the β subunit.

On the other hand, in mammalian cultured neurons it has been proposed that a complex formed by the scaffold proteins LIN-2/CASK, LIN-10/MINT and LIN-7 is involved in the localization of VGCCs at the synapse^35^ and that SAP97 forms a complex with CASK^36^. The association of DLGS97 with LIN7 has been reported in the postsynaptic compartment in the Drosophila NMJ^37^. Furthermore, an association between the L27 domain of DLGS97 and the L27 domain of Drosophila CASK has been shown *in vitro*^36^, however there are no reports of this type of association with DLGA. Another protein involved in the localization of calcium channels in the active zone is RIM^38^. *Drosophila rim* has been involved in synaptic homeostasis and the modulation of vesicle pools. Surprisingly *rim* mutants, display low probability of release and altered responses to different calcium concentrations^39^. Recently it was shown that *spinophilin* mutants display a phenotype with bigger quantal size and GLUR fields size with a higher proportion of GLURIIA subtype of receptors as well as decreased EJCs and decreased pair pulse facilitation^40^. This is a phenotype very similar to the one described here for *dlg* mutants. The authors in this report did not explore the calcium channels abundance or distribution and here we did not explore the link of DLG to Neuroligins, Neurexins and Syd. It would be interesting to determine if there is a link between Spinophilin and DLG.

Taken together these results show that *dlgS97* is the main isoform responsible for the postsynaptic defects in the *dlg*^*XI-2*^ mutants; which comprise the increase in the size of the receptors fields and the change in the ratio of GLURIIA/GLURIIB. Our results as well support a model in which DLG forms a presynaptic complex that includes Cacophony where the absence of either form of DLG leads to defects in the localization of the voltage dependent calcium channel and to a decrease in the entrance of calcium to the bouton; which in turn affect the probability of release and the short-term plasticity in the mutants. The results described here highlight the specificity of the function of DLGS97 and DLGA proteins and describe for the first time an *in vivo* presynaptic role of DLG proteins.

## Materials and Methods

### Drosophila stocks and genetics

The following strains were used: Canton S; *dlg*^*XI-2*^; *dlgS97*^*5*^; *dlgA*^*40.2*^; *UAS-dlgA-EGFP*; *UAS-dlgS97-EGFP*^7^,^41^; *UAS-CAC1-EGFP* (*422A*) *and* Df(1)BSC288,w{1118}/BinSincy (Bloomington Stock Center, Bloomington, IN*); UAS-RNAi-dlg1*^42^ (VDRC 41134, Vienna Drosophila Research Center, Vienna, Austria); the motoneuron activator strain *OK6-GAL4* and the muscle activator strain *C57-GAL4* (kindly provided by Dr. Ulrich Thomas), All flies were stored and crossed at 25 °C.

### Immunohistochemistry

Male larval body wall muscle preparations were stained as described previously^12^,^41^ for CAC1 immunostaining we proceed as described in ref. 31. Primary antibodies used were: rabbit anti-glutamate receptor III (GLURIII) (1:200) (kindly provided by Dr. Tobias Rasse and Dr. David Featherstone), rabbit anti- glutamate receptor IIB (GLURIIB) (1:300, gently provided by Dr. David Featherstone), mouse anti GLURIIA (8B4D2, 1:800, C. Goodman) and mouse anti-Brp (nc82; 1:200, E. Buchner) purchased from Developmental Studies Hybridoma Bank; Cy-5 conjugated anti-HRP and HRP- or fluorescent-coupled secondary antibodies (1:200; Jackson ImmunoResearch, West Grove, PA) and mouse anti-GFP 3E6 (1:200, Invitrogen, West Grove, PA). After immunocytochemical procedures samples were mounted in Vectashield mounting medium (Vector Laboratories, Burlingame, CA). Images were captured using a confocal microscope (Olympus FV1000), deconvolved using Huygens Software (Scientific Volume Imaging, Hilversum, The Netherlands) and quantified using ImageJ (U. S. National Institutes of Health). Data was collected in Excel (Microsoft) to be processed. Each data point corresponds to the average quantification of the clusters of at least three boutons from two pictures per larva. The number of data corresponds to the number of larvae imaged.

### Electrophysiology

Postsynaptic currents from male larvae of the specified genotypes were recorded at segment A3 of ventral longitudinal muscle 6 in third instar larva using two-electrode voltage clamp (OC-725C, Harvard Apparatus, Holliston, MA) at a −80 mV holding potential in modified HL3.1 solution (in mM: 10 NaHCO_3_, 5 KCl, 4 MgCl, 5 HEPES, 70 NaCl, 5 Trehalose, 115 Sucrose, pH 7.2). Final Ca^2+^ concentration was adjusted to the desired level indicated in each figure legend. Miniature currents were recorded in the presence of saxitoxin (SXT 100 nM, gift from Dr. Néstor Lagos, Universidad de Chile) to block neuronal action potentials. Data acquisition and analysis was performed using Axoscope 9.0 and Clampfit 9.0 software (Molecular Devices, Sunnyvale, CA). Quantal content was estimated by dividing the current peak of nerve-evoked currents by the peak from mEJCs distribution histograms for each genotype. Motor nerves innervating the musculature were severed and placed into a suction electrode so action potential stimulation could be applied at the indicated frequencies using a programmable stimulator (Master8, AMPI, Jerusalem, Israel).

### Immunoprecipitation

WT and Elav-CAC1-GFP flies of either sex were collected, frozen with liquid nitrogen and pass through sieves in order to collect the heads. Heads were lysed with RIPA buffer (1X) and used 5000 μg of protein per each immunoprecipitation; inputs were 10%. The head homogenize was mixed with anti-GFP conjugated beads (Chromoteck; Planegg-Martinsried, Germany) (25 μl of GFP-conjugated beads per reaction) and RIPA buffer up to 1 mL total volume; the samples were incubated for 2 hrs. in a rotator at 4 °C and were washed 4 times with RIPA Buffer for 10 min each at 4 °C. The elution was carried out with 20 μL of Laemmeli buffer (1X) for 15 min, the samples were loaded in an 8% SDS page, transferred for 1 hour to nitrocellulose and the blot analyzed using rabbit anti GFP (Invitrogen, 1:1000) and mouse anti-DLG_PDZ_ (4F3, Developmental Studies Hybridoma Bank, 1:1000) followed by anti mouse and rabbit antibodies coupled to HRP. The blot was developed by chemiluminescence detection (ECL, Amersham).

### Calcium imaging

Images were acquired in an Olympus (Center Valley, PA, USA) BX61WI coupled to a CCD camera Hamamatsu ORCA-*R*^*2*^ controlled by Cell^R software (Olympus, Tokyo, Japan), with an objective lens 60X LUM PlanFl water immersion with a numerical aperture of 0.9. After acquiring a 10–20 s base line at 10 Hz, KCl 1 M puff of 50–300 ms was delivered by picospitzer Narishige (Japan), using the same pipette for control paired batch experiments. Recorded images were processed using the following formula: (∆F/F_0_ = F − F_bkg_/F_0_ − F_bkg_), where F is the fluorescence intensity measured in type Ib boutons of muscle 6/7, F_bkg_ is the background signal, and F_0_ is the intensity at the initial time. The rise time was calculated by fitting [1 − Exp(−t/b)], where b is the time to reach the 63% of the maximum intensity and t is time. Decay time was fitted to a single exponential decay from peak value. Images were analyzed using ImageJ software (NIH, Bethesda, Maryland, USA).

### Statistical Analysis

The fitting of the calcium curves was performed using Origin Software (Origin Lab Corporation). Data from Clampfit or Excel was exported to GraphPad Prism 5 (GraphPad Software, Inc., La Jolla, CA) for statistical analysis. Statistical significance was determined using ANOVA followed by Tukey test.

## Additional Information

**How to cite this article**: Astorga, C. *et al*. Presynaptic DLG regulates synaptic function through the localization of voltage-activated Ca^2+^ Channels. *Sci. Rep*. **6**, 32132; doi: 10.1038/srep32132 (2016).

## Supplementary Material

Supplementary Information
