# Supplementary material for: Presynaptic DLG regulates synaptic function through the localization of voltage-activated Ca2+ Channels
Source: Sci Rep. 2016 Aug 30;6:32132. doi: 10.1038/srep32132 (PMC5004195; doi:10.1038/srep32132)
Supplement: Supplementary Information [file srep32132-s1.pdf]

Presynaptic DLG regulates synaptic function through the localization of voltage-activated Ca^{2+} Channels.

Abbreviated title: Differential roles of *Drosophila* DLG in pre and post-synaptic function

César Astorga[1], Ramón A. Jorquera[2, 3], Mauricio Ramírez[1], Andrés Kohler[1], Estefanía López[1], Ricardo Delgado[4], Alex Córdova[1], Patricio Olguín[1,5] and Jimena Sierralta[1,2,*].

1. Biomedical Neuroscience Institute, Faculty of Medicine, Universidad de Chile; Santiago, Chile.
2. Program of Physiology and Biophysics, Institute of Biomedical Sciences, Faculty of Medicine, Universidad de Chile, Santiago, Chile.
3. Neuroscience Department, School of Medicine, Universidad Central del Caribe, Bayamon, 00956, PR, USA.
4. Department of Biology, Faculty of Science, Universidad de Chile, Santiago, Chile.
5. Program of Human Genetics, Institute of Biomedical Sciences, Faculty of Medicine, Universidad de Chile, Santiago, Chile.

*** Corresponding author: Jimena Sierralta. email:** jimena@neuro.med.uchile.cl

Address: Independencia 1027, Santiago, Chile 8380453

Supplementary Figures

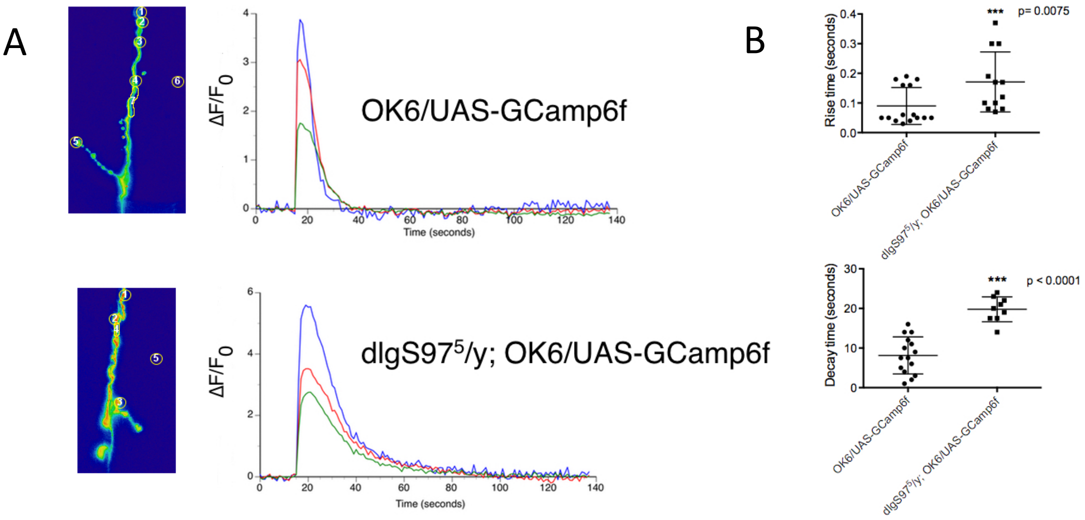

Figure S1: **Intracellular calcium increase in response to stimulus is slower in dlgS97 mutants**. A) Representative recording of the changes in fluorescence (of GCamp6f expressed in the motoneuron using OK6-Gal4. The calcium increase was induced with potassium delivered locally using the same conditions for all larvae tested. At the right is depicted the image of the branch where the ROIs are labeled, for simplicity only three of the boutons are shown in the graph. B) Quantification of the average rise (upper graph) and decay (bottom graph) time in control and *dlgS97* mutant larvae (n=3 larvae, 2-5 boutons per larva).

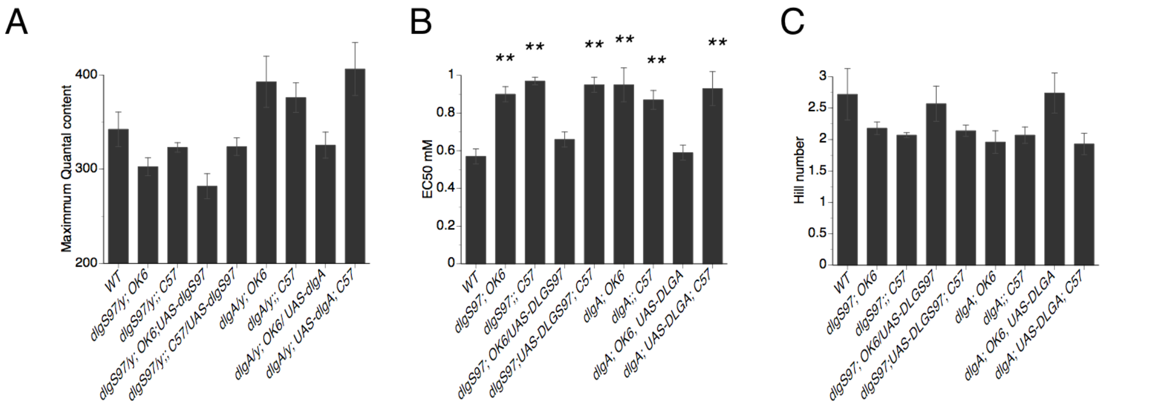

Figure S2: **Parameters of the calcium curves shown in Figure 6**. A) Maximal quantal contents of the Hill curves fitted to the data obtained for each genotype as shown in Figure 6D and 6E. B) EC50 obtained from the normalized calcium curves shown in Figure 6D an 6E. C) Hill number obtained for the fitting for each genotype. Only DLG proteins expressed presynaptically were able to restore the WT parameters in the mutant lines. ** p<0.01
